# Particle swarm optimization based analysis to unlocking the neutrino mass puzzle using $SU(2)_L \times U(1)_Y \times A_{4}\times S_2\times Z_{10} \times Z_{3}$ flavor symmetry

**DOI:** 10.1038/s41598-024-81791-3

**Published:** 2025-02-11

**Authors:** Muhammad Waheed Aslam, Abrar Ahmad Zafar, Muhammad Naeem Aslam, Abdul Aziz Bhatti, Talab Hussain, Munawar Iqbal, Mohammed S. Abdo

**Affiliations:** 1 Department of Physics, University of the Punjab, Lahore, Pakistan; 2 Department of Mathematics, Lahore Garrison University, Lahore, Pakistan; 3 Centre for High Energy Physics, University of the Punjab, Lahore, Pakistan; 4 College of Statistical Sciences, University of the Punjab, Lahore, Pakistan; 5 Department of Mathematics, Hodeidah University, P.O. Box 3114, Al-Hudaydah, Yemen

**Keywords:** Discrete symmetry, Neutrino mixing, Particle swarm optimization, Phenomenology, Theoretical particle physics

## Abstract

New research has highlighted a shortfall in the Standard Model (SM) because it predicts neutrinos to have zero mass. However, recent experiments on neutrino oscillation have revealed that the majority of neutrino parameters indeed indicate their significant mass. In response, scientists are increasingly incorporating discrete symmetries alongside continuous ones for the observed patterns of neutrino mixing. In this study, we have examined a model within $SU(2)_L \times U(1)_Y \times A_{4}\times S_2\times Z_{10} \times Z_{3}$ symmetry to estimate the neutrino masses using particle swarm optimization technique for both mass hierarchy of neutrino. This model employed a hybrid seesaw mechanism, a combination of seesaw mechanism of type-I and type-II, to establish the effective Majorana neutrino mass matrix. After calculating the mass eigenvalues and lepton mixing matrix upto second order perturbation theory in this framework, this study seeks to investigate the scalar potential for vacuum expectation values (VEVs), optimize the parameters, $U_{PMNS}$ matrix, neutrino masses: ${m_{1}^{\prime }}^{(N)}(upper)=4.0000 \times 10^{-2}\ eV,$ ${m_{2}^{\prime }}^{(N)}(upper)=4.0000 \times 10^{-2}\ eV,$ ${m_{3}^{\prime }}^{(N)}(upper)=4.0000 \times 10^{-2}\ eV,$ ${m_{1}^{\prime }}^{(I)}(upper)=3.8628\times 10^{-2}\ eV,$ ${m_{2}^{\prime }}^{(I)}(upper)=4.0548\times 10^{-2}\ eV,$ ${m_{3}^{\prime }}^{(I)}(upper)=3.8532\times 10^{-2}\ eV,$ ${m_{1}^{\prime }}^{(N)}(lower)=2.0000 \times 10^{-2}\ eV,$ ${m_{2}^{\prime }}^{(N)}(lower)=2.0000 \times 10^{-2}\ eV,$ ${m_{3}^{\prime }}^{(N)}(lower)=2.0000 \times 10^{-2}\ eV,$ ${m_{1}^{\prime }}^{(I)}(lower)=1.1049\times 10^{-2}\ eV,$ ${m_{2}^{\prime }}^{(I)}(lower)=3.9298\times 10^{-2}\ eV$ and ${m_{3}^{\prime }}^{(I)}(lower)=9.6381\times 10^{-3}\ eV,$ effective neutrino mass parameters: $\langle {m_{ee}} \rangle ^{N}(upper)=40.0050 \ meV,$ $\langle {m_{\beta }} \rangle ^{N}(upper)=40.0025\ meV,$ $\langle {m_{ee}} \rangle ^{I}(upper)=39.2181\ meV,$ $\langle {m_{\beta }} \rangle ^{I}(upper)=39.2257\ meV,$ $\langle {m_{ee}} \rangle ^{N}(lower)=20.0024\ meV,$ $\langle {m_{\beta }} \rangle ^{N}(lower)=20.0012\ meV,$ $\langle {m_{ee}} \rangle ^{I}(lower)=19.6608\ meV,$ $\langle {m_{\beta }} \rangle ^{I}(lower)=23.5908\ meV,$ are predicted for both mass hierarchy through particle swarm optimization (PSO), showing strong agreement with recent experimental findings. The Dirac CP-violating phase $\delta$ is measured to be $-\pi /2$.

## Introduction

Known as the “ghost particles” of the universe, neutrinos have long intrigued the interest of cosmologists and physicists alike. Despite being among the most prevalent particles in the universe, these elementary particles (which are electrically neutral and almost massless) interact with matter very weakly, which makes them notoriously difficult to detect. In 1930, Wolfgang Pauli postulated the existence of neutrinos as a possible explanation for the violation of energy conservation observed in beta decay, they were only ever considered theoretical particles. Frederick Reines and Clyde Cowan eventually detected neutrinos in 1956^1^. Neutrinos, in spite of their spectral appearance, are essential to the understanding of fundamental physics and the universe’s evolution. Many astrophysical processes, such as nuclear fusion in stars^2^, supernova explosions^3–7^, and even the Big Bang itself^8,9^, produce them. Neutrinos are also essential for solving some of the most significant mysteries in cosmology and particle physics, including the properties of the elusive Higgs boson^10^, the nature of dark matter^11–15^, and the universe’s imbalance between matter and antimatter^16^. One of the central puzzles surrounding neutrinos is their masses^17^. Neutrinos were originally thought to be massless in accordance with the SM of particle physics^18^. However, experiments in the late 20th and early 21st centuries, such as those conducted by the Super-Kamiokande^19^, KamLAND^20^, K2K^21^, Fermilab-MINOS^22^, Sudbury Neutrino Observatory collaborations^19^ and CERN-OPERA^23^ provided irrefutable evidence that neutrinos oscillate between different flavors (electron, muon, and tau), a phenomenon that can only occur if they possess non-zero masses. This discovery fundamentally challenged our understanding of neutrinos and underscored the need for new theoretical frameworks beyond the SM.

Researchers are looking in detail into seesaw frameworks, particularly type-I^24–28^ and type-II^29–31^, aside from several other methods to explain small neutrino masses. Majorana and Dirac mass terms derive from the introduction of extra right-handed neutrinos in SM in type I. Majorana mass terms derive from the introduction of heavy $SU(2)_L$ triplet in SM in type-II. A hybrid seesaw mechanism^24,32,33^ has been proposed for improved mass suppression and new mixing patterns by combining type-I and type-II. With this hybrid technique, one may explore various lepton mixing scenarios and generate effective Majorana neutrino mass matrices.

Considering the recent discovery of non-zero, small neutrino masses in multiple neutrino oscillation experiments, numerous models for neutrino mass have been developed. These models are constructed based on different discrete symmetries such as $S_4$, $A_4$, $\Delta (27)$ and $T_7$ etc.^34–60^. In most cases, models extending the Standard Model (SM) incorporate symmetries through the addition of specific field contents with their respective charges. These models predict distinctive neutrino masses and mixing patterns by employing different seesaw mechanisms (Type I, II, and III). However, the current model introduces a new aspect by using a hybrid seesaw mechanism combining Type I and Type II, which is not commonly explored in earlier studies focusing on $A_4$ symmetry. This hybrid approach offers a novel pathway to derive neutrino masses and mixing parameters, contributing to the uniqueness of the model compared to previous work.

Despite this innovation, deriving analytical expressions in such models remains challenging due to the complexity of the equations involved. These equations often contain nonlinear terms relating to the neutrino mass eigenstates, making them difficult to solve without advanced mathematical techniques. Compared to earlier $A_4$ models, the current model tackles these challenges with computational methods, particularly using particle swarm optimization (PSO), which proves to be highly effective for solving such complex optimization problems. The collective actions of fish and birds serve as the model for this population-based algorithm and metaheuristic approach. PSO is used for approximating parameters in different types of research problems^61,62^. In 1995, Russell Eberhart and James Kennedy introduced the concept of PSO.^63–65^, drawing inspiration from genetic algorithms (GAs) to refine its design^63^. PSO is commonly used to find optimal solutions to optimization problems, where the aim is to minimize or maximize a particular fitness function. PSO is versatile and has been utilized in diverse optimization scenarios, including engineering design^66–68^, image processing^69–71^, financial modeling^72–75^ and neural network training^76,77^. Its efficacy is further underscored by its widespread use in diverse optimization challenges, encompassing high-dimensional data clustering^78,79^, parameter estimation for chaotic maps^80,81^, optimization of core loading models in nuclear reactors^82^, optimization of nonlinear reference frames^83^, attainment of optimal reactive power distribution^84^, as well as problem-solving in domains such as optical properties of multilayer thin films^85–88^ and autoregressive models with moving average^89–91^. Additionally, PSO has proven effective in addressing challenges related to parameter estimation in electromagnetic plane waves^92^. Its simplicity, ease of implementation, and ability to handle non-linear and complex objective functions make it a popular choice for solving optimization problems. PSO stands out in particular for having an easy-to-implement architecture and requiring less memory^93,94^.

After calculating the mass eigenvalues and lepton mixing matrix upto second order perturbation theory in the framework^95^ based on $A_{4}$ symmetry, this study seeks to investigate the minimization of the scalar potential for VEVs and optimize the parameters for $U_{PMNS}$ matrix, neutrino masses and effective neutrino mass parameters: $\langle m_{ee}\rangle ,\ m_{\beta }$, for both mass hierarchy through particle swarm optimization (PSO). The format of this article is as follows: The $A_4$ model is presented in the next section 2. In addition to describe the superpotential terms for charged leptons and neutrinos, “Mass matrices of charged lepton and neutrino” section provide the explanation for the mass eigenvalues and mixing matrix upto second order perturbation theory. “Numerical analysis” section focuses on the utilization of PSO to determine optimal parameter values for computing neutrino masses. “Vacuum alignment studies” section presents the scalar potential invariant under $SU(2)_L \times U(1)_Y \times A_{4}\times Z_{10} \times Z_{3}$, along with conditions for its minimization and explores the utilization of PSO in determining optimal parameter values for VEVs of the scalars with addition of $S_2$ flavour symmetry. At the end, in section 5, we provide a conclusion of our research.

## The $A_4$ based model

In reference^95^, the authors extended the SM group with $A_4$ symmetry with three right handed heavy singlet neutrino fields $(\nu _{e_R} ,\nu _{\mu _R} ,\nu _{\tau _R})$ and with seven scalars $\phi$, $\Phi$, $\eta$, $\kappa$, $\Delta$, $\xi$, $\xi ^{\prime }$. The $SU(2)_L$ doublets $\phi$, $\Phi$ and $SU(2)_L$ triplet $\Delta$ are taken as $A_4$ triplet. Four $SU(2)_L$ singlets $\eta$, $\kappa$, $\xi$ and $\xi ^{\prime }$ are taken as the singlets of $A_4$ as $1^{\prime \prime }$, $1^{\prime }$, 1 and 1 respectively. A additional symmetry, namely $Z_{10}$ is also introduced to incorporate the undesired terms, where, $Z_{10}$ refers to the symmetry of integers modulo 10. A summary of all the fields under $SU(2)_L$, $A_4$, $Z_{10}$, $Z_{3}$ and $U(1)_Y$ are shown in Table 1.

**Table 1:** The properties of transformation under $SU(2)_L \times U(1)_Y \times A_{4}\times Z_{10} \times Z_{3}$.

Fields	$D_{l_{L}}$	$l_R$	$\nu _{l_{R}}$	$\phi$	$\Phi$	$\eta$	$\kappa$	$\Delta$	$\xi$	$\xi ^{\prime }$
$SU(2)_L$	2	1	1	2	2	1	1	3	1	1
$A_4$	3	$(1, 1^{\prime \prime }, 1^{\prime })$	$(1, 1^{\prime \prime }, 1^{\prime })$	3	3	$1^{\prime \prime }$	$1^{\prime }$	3	1	1
$U(1)_Y$	$-1$	$-2$	0	1	$-1$	0	0	$-2$	0	0
$Z_{10}$	0	0	(0, 4, 6)	0	0	2	8	0	6	4
$Z_{3}$	1	$(\omega ,\omega ,\omega )$	(1, 1, 1)	$\omega ^2$	1	1	1	1	1	1

We also incorporate an additional $S_2$ symmetry. The $S_2$ group, known as the permutation group of two elements, has specific generators when expressed in the 3-dimensional representation, which are as follows:

$$\begin{aligned} e = \begin{pmatrix} 1 & 0 & 0 \\ 0 & 1 & 0 \\ 0 & 0 & 1 \end{pmatrix}, \quad a = \begin{pmatrix} 1 & 0 & 0 \\ 0 & 0 & 1 \\ 0 & 1 & 0 \end{pmatrix} \end{aligned}$$

By choosing the following couplings and imposing $S_2$ flavor symmetry, the resulting constraints are as follows:

$$\begin{aligned} y_2 \leftrightarrow y_3,\quad y_{R_1} \leftrightarrow y_{R_2}, \quad \xi \leftrightarrow \xi ', \quad \eta \leftrightarrow \kappa \end{aligned}$$

All remaining fields transform trivially under $S_2$.

### Mass matrices of charged lepton and neutrino

The Lagrangian serves as a cornerstone in describing the interactions and behaviors of particles within the context of physics of particles. The superpotential term for charged leptons, Dirac neutrinos and right handed Majorana neutrinos is given as

1 $$\begin{aligned} \begin{aligned} - {\mathcal {L}}_Y&= y_e ({\bar{D}}_{l_L}\phi )_{1}\,e_{R} + y_{\mu } ({\bar{D}}_{l_L}\phi )_{1^{\prime }} \,\mu _{R}+\,y_{\tau } ({\bar{D}}_{l_L}\phi )_{1^{\prime \prime }} \tau _{R}+ y_1\,({\bar{D}}_{l_L}\Phi )_1\,\nu _{e_R} \,\\ &+ \frac{y_2}{\Lambda }\,({\bar{D}}_{l_L}\Phi )_{1^{\prime }} \,\nu _{{\mu }_{R}}\,\xi + \frac{y_3}{\Lambda }\,({\bar{D}}_{l_L}\Phi )_{1^{\prime \prime }}\nu _{{\tau }_{R}}\,\xi ^{\prime } +\,\frac{1}{2}\,M_1\,({\nu ^c_{e_R}}\,\nu _{e_R}) +\,\frac{1}{2}\,M_2\,[({\nu ^c_{\mu _{R}}}\nu _{\tau _{R}})\\ &\,+ ({\nu ^c_{\tau _{R}}} \nu _{\mu _{R}})]+\,\frac{1}{2}\,y_{R_1}({\nu ^c_{\mu _{R}}}\,\nu _{\mu _{R}})\,\eta +\frac{1}{2}\,y_{R_2}({\nu ^c_{\tau _R}}\nu _{\tau _{R}})\,\kappa +\, y({\bar{D}}_{l_L}\, D_{l_L}^c)\,{i \sigma _2 \Delta } \,+ h.c., \end{aligned} \end{aligned}$$

In this context, $y_e$, $y_{\mu }$, $y_{\tau }$, $y_{1,2,3}$, $M_{1,2}$, $y_{R_{1,2}}$ and *y* are represent the dimensionless Yukawa couplings. Due to the VEVs (see section 4) and $S_2$ flavour symmetry, one can generate mass matrices for charged leptons ($M_l$), Dirac neutrinos ($M_D$) and right handed Majorana neutrinos as

2 $$\begin{aligned} & \begin{aligned}&M_{l}=v \begin{pmatrix} y_{e} & 0 & 0\\ 0 & y_{\mu } & 0\\ 0 & 0 & y_{\tau }\\ \end{pmatrix} \end{aligned}, \qquad \begin{aligned} M_{D}=u \begin{pmatrix} 0 & \frac{y_{2}v_\epsilon }{\Lambda } & \frac{y_{2}v_\epsilon }{\Lambda }\\ y_{1} & 0 & \frac{y_{2}v_\epsilon }{\Lambda }\\ y_{1} & \frac{y_{2}v_\epsilon }{\Lambda } & 0\\ \end{pmatrix} \end{aligned}, \end{aligned}$$

3 $$\begin{aligned} & \begin{aligned} M_{R}= \begin{pmatrix} M_1 & 0 & 0\\ 0 & y_Rv_m & M_2\\ 0 & M_2 & y_Rv_m\\ \end{pmatrix}, \end{aligned} \qquad \begin{aligned} M^{\prime \prime }= \frac{y\omega }{3} \begin{pmatrix} 0 & 1 & -1\\ 1 & 2 & 0\\ -1 & 0 & -2\\ \end{pmatrix}. \end{aligned} \end{aligned}$$

Here, seesaw frameworks, particularly type-I^24–28^ and type-II^29–31^, used besides several other methods to explain small neutrino masses. Majorana and Dirac mass terms derived from the introduction of extra right-handed neutrinos in SM in type I. Majorana mass terms ($M^{\prime \prime }$) derived from the introduction of heavy $SU(2)_L$ triplet in SM in type-II. In other words, a hybrid seesaw mechanism^24,32,33^ proposed for improved mass suppression and new mixing patterns by combining type-I and type-II. With this hybrid technique, one may explore various lepton mixing scenarios and generate effective Majorana neutrino mass matrices $(M_\nu )$ as

4 $$\begin{aligned} \begin{aligned} M_\nu =M^{\prime }+M^{\prime \prime }=-m_D M_R^{-1} m_D^{T}+M^{\prime \prime } \end{aligned} \end{aligned}$$

with

5 $$\begin{aligned} \begin{aligned} M^{\prime }&= \begin{pmatrix} P & Q & Q\\ Q& R & S\\ Q & S & R \end{pmatrix}, \qquad M^{\prime \prime }=\begin{pmatrix} 0 & p & -p\\ p & q & 0\\ -p & 0 & -q\\ \end{pmatrix},\\ p&=\frac{y\omega }{3},\quad q=2p,\quad P=-\frac{2 u^2 {v_\epsilon }^2 {y_2}^2}{\Lambda ^2 (M_2+v_m y_R)}, \quad Q=-\frac{u^2 {v_\epsilon }^2 {y_2}^2}{\Lambda ^2 (M_2+v_m y_R)},\quad \\ R&=u^2 \left( \frac{v_m {v_\epsilon }^2 {y_2}^2 y_R}{\Lambda ^2 \left( {M_2}^2-{v_m}^2 {y_R}^2\right) }-\frac{{y_1}^2}{{M_1}}\right) , \quad S=u^2 \left( -\frac{{y_1}^2}{{M_1}}-\frac{{M_2} {v_\epsilon }^2 {y_2}^2}{\Lambda ^2 \left( {M_2}^2-{v_m}^2 {y_R}^2\right) }\right) . \end{aligned} \end{aligned}$$

The first effective Majorana matrix is diagonalized under the conditions $y_2=y_3$, $y_{R_1}=y_{R_2}$ and $S_2$ symmetry, by the subsequent mixing matrix,

6 $$\begin{aligned} \begin{aligned} U_0= \begin{pmatrix} c & s & 0\\ -s/\sqrt{2}& c/\sqrt{2} & 1/\sqrt{2}\\ -s/\sqrt{2} & c/\sqrt{2} & -1/\sqrt{2} \end{pmatrix}, \end{aligned} \end{aligned}$$

such as $diag(m_1,\ m_2,\ m_3)={U_0}^T M_{1}\ {U_0}$, where, $c=\cos \theta$, $s=\sin \theta$ and $\theta =arccos(\frac{k}{\sqrt{k^2+2}})$ with,

7 $$\begin{aligned} \begin{aligned} k=\frac{P-R-S-\sqrt{P^2-2 P R-2 P S+8 Q^2+R^2+2 R S+S^2}}{2 Q} \end{aligned}, \end{aligned}$$

and

8 $$\begin{aligned} \begin{aligned} m_{1,2}=\frac{1}{2} (P+R+S\mp \sqrt{(-P+R+S)^2+8 Q^2}), \qquad m_3=R-S. \end{aligned} \end{aligned}$$

In the context of three-neutrino physics, the mixing matrix of lepton $(U_{PMNS})$ may be represented as^96^

9 $$\begin{aligned} \begin{aligned} U_{PMNS}= \begin{pmatrix} c_{12}c_{13} & s_{12}c_{13} & s_{13} e^{i\delta }\\ -s_{12}c_{23}-c_{12}s_{13}s_{23}e^{i\delta } & c_{12}c_{23}-s_{12}s_{13}s_{23}e^{i\delta } & c_{13}s_{23}\\ s_{12}s_{23}-c_{12}c_{23}s_{13}e^{i\delta } & -c_{12}s_{23}-s_{12}s_{13}c_{23}e^{i\delta } & c_{13}c_{23} \end{pmatrix} P_{12} \end{aligned} \end{aligned}$$

where, $P_{12}=diag(1, e^{i\beta _{1}}, e^{i\beta _{2}})$ which contains two Majorana phases that do not influence neutrino oscillations. The matrix $U_0$ in Eq. (6) suggests $\theta _{23} = \pi /4$, $\theta _{13} = 0$ and $\theta _{12} = \theta$, Recent data contradicts this claim. However, the inclusion of the second matrix in Eq. (4) is expected to ameliorate this discrepancy. In first-order perturbation corrections, the second matrix in Eq. (4) doesn’t affect the eigenvalues but it does influence the eigenvectors. Moving to second-order perturbation theory, this matrix contributes to the determination of both eigenvalues and eigenvectors. Consequently, the masses of neutrino upto the second order perturbation corrections can be expressed as:

10 $$\begin{aligned} \begin{aligned} m^{\prime }_1=m_1+\frac{p^2{\Gamma _1}^2}{m_1-m_3},\quad m^{\prime }_2=m_2+\frac{p^2{\Gamma _2}^2}{m_2-m_3},\quad m^{\prime }_3=m_3+p^2\left( \frac{2{\Gamma _3}^2}{m_3-m_1}+\frac{{\Gamma _2}^2}{m_3-m_2}\right) , \end{aligned} \end{aligned}$$

where the parameters *p*, *q*, and $m_{1,2,3}$ are defined in Eqs. (5) and (8), respectively. The contributions to the eigenvalues from first-order perturbation are negligible, with only minor changes arising at second-order perturbation. In Eq. (10), the terms $\frac{p^2{\Gamma _1}^2}{m_1-m_3}$, $\frac{p^2{\Gamma _2}^2}{m_2-m_3}$, and $p^2\left( \frac{2{\Gamma _3}^2}{m_3-m_1}+\frac{{\Gamma _2}^2}{m_3-m_2}\right)$ are significantly smaller compared to $m_1$, $m_2$, and $m_3$, respectively. This is consistent with the condition $M^{\prime \prime } \ll M^{\prime }$. Subsequently, the resulting lepton mixing matrix is as follows:

11 $$\begin{aligned} \begin{aligned} U=U_0+\Delta U+\Delta U^{\prime } \end{aligned} \end{aligned}$$

where $U_0$ is given by in Eq. (6), $\Delta U$ represents the mixing matrix corresponding to first-order corrections, and $\Delta U^{\prime }$ represents the mixing matrix corresponding to second-order corrections. They have the following entries:

$$\begin{aligned} \begin{aligned} (\Delta U)_{11}&=(\Delta U)_{12}=0, \end{aligned} \end{aligned}$$

$$\begin{aligned} \begin{aligned} (\Delta U)_{13}&=p\left( \frac{\Gamma _1 \cos {\theta }}{{m_3}-{m_1}}+\frac{\Gamma _2\sin {\theta }}{{m_3}-{m_2}}\right) , \end{aligned} \end{aligned}$$

$$\begin{aligned} \begin{aligned} (\Delta U)_{21}=-(\Delta U)_{31}=\frac{p\Gamma _3}{{m_3}-{m_1}},\quad (\Delta U)_{32}=-(\Delta U)_{22}=\frac{\sqrt{2}\Gamma _2 p}{2({m_3}-{m_2})}, \end{aligned} \end{aligned}$$

$$\begin{aligned} \begin{aligned} (\Delta U)_{23}&=(\Delta U)_{33}=\frac{p \left( ({m_1}-{m_2})\Gamma _4+2 ({m_1}+{m_2}-2 {m_3})\right) }{2 \sqrt{2} ({m_3}-{m_1}) ({m_2}-{m_3})}, \end{aligned} \end{aligned}$$

$$\begin{aligned} \begin{aligned} (\Delta U^{\prime })_{11}=\frac{p^2\Gamma _1}{2 ({m_1}-{m_3})^2} \biggl [-\cos \theta \Gamma _1+\frac{2 \sin \theta ({m_1}-{m_3})\Gamma _2}{{m_1}-{m_2}}\biggl ], \end{aligned} \end{aligned}$$

$$\begin{aligned} \begin{aligned} (\Delta U^{\prime })_{12}&=\frac{p^2\Gamma _2}{2 ({m_2}-{m_3})^2}&\biggl [-\sin \theta \Gamma _2 -\frac{2 \cos \theta ({m_2}-{m_3})\Gamma _1}{{m_1}-{m_2}}\biggl ], \quad (\delta U^{\prime })_{13}=0, \end{aligned} \end{aligned}$$

$$\begin{aligned} \begin{aligned} (\Delta U^{\prime })_{21}=(\Delta U^{\prime })_{31}=p^2 \Gamma _1&\biggl [\frac{\Gamma _5 (3 {m_1}-{m_2}-2 {m_3})+{m_1}+{m_2}-2 {m_3})}{{2 \sqrt{2} ({m_1}-{m_2}) ({m_1}-{m_3})^2} }\biggl ], \end{aligned} \end{aligned}$$

$$\begin{aligned} \begin{aligned} (\Delta U^{\prime })_{22}=(\Delta U^{\prime })_{32}=-p^2 \Gamma _2\biggl [\frac{\Gamma _5 ({m_1}-3 {m_2}+2 {m_3})+{m_1}+{m_2}-2 {m_3}}{2 \sqrt{2} ({m_1}-{m_2}) ({m_2}-{m_3})^2}\biggl ], \end{aligned} \end{aligned}$$

$$\begin{aligned} \begin{aligned} (\Delta U^{\prime })_{23}=&-(\Delta U^{\prime })_{33}=\frac{-p^2}{2 \sqrt{2}}\biggl [\frac{{\Gamma _1}^2}{({m_1}-{m_3})^2}+\frac{{\Gamma _2}^2}{({m_2}-{m_3})^2}\biggl ], \end{aligned} \end{aligned}$$

with, $\Gamma _1=-2\sin {\theta }+\sqrt{2}\cos {\theta }$, $\Gamma _2=\sqrt{2}\sin {\theta }+2\cos {\theta }$, $\Gamma _3=\sqrt{2}\sin {\theta }-\cos {\theta }$, $\Gamma _4=\sqrt{2}\sin {2\theta }+2\cos {2\theta }$ and $\Gamma _5=\cos {2\theta }+\sqrt{2}\sin {\theta }\cos {\theta }$. The lepton mixing angles can be determined from Eqs. (9) and (11), which define the mixing matrix of neutrino:

12 $$\begin{aligned} \begin{aligned} t_{12}=\frac{|U_{12}|}{|U_{11}|},\quad t_{23}=\frac{|U_{23}|}{|U_{33}|}, \quad s_{13}=|U_{13}|, \end{aligned} \end{aligned}$$

with, $s_{ij}=\sin {\theta _{ij}}$, $c_{ij}=\cos {\theta _{ij}}$ and $t_{ij}=\tan {\theta _{ij}}$. Most of $A_4$ models predict $\theta _{13}=0$. In this model, there is a deviation, for sure there is some correlation with other observable like $\theta _{12}$, $\theta _{23}$. The correlations between different parameters are given in Fig. 1.

**Fig. 1:**
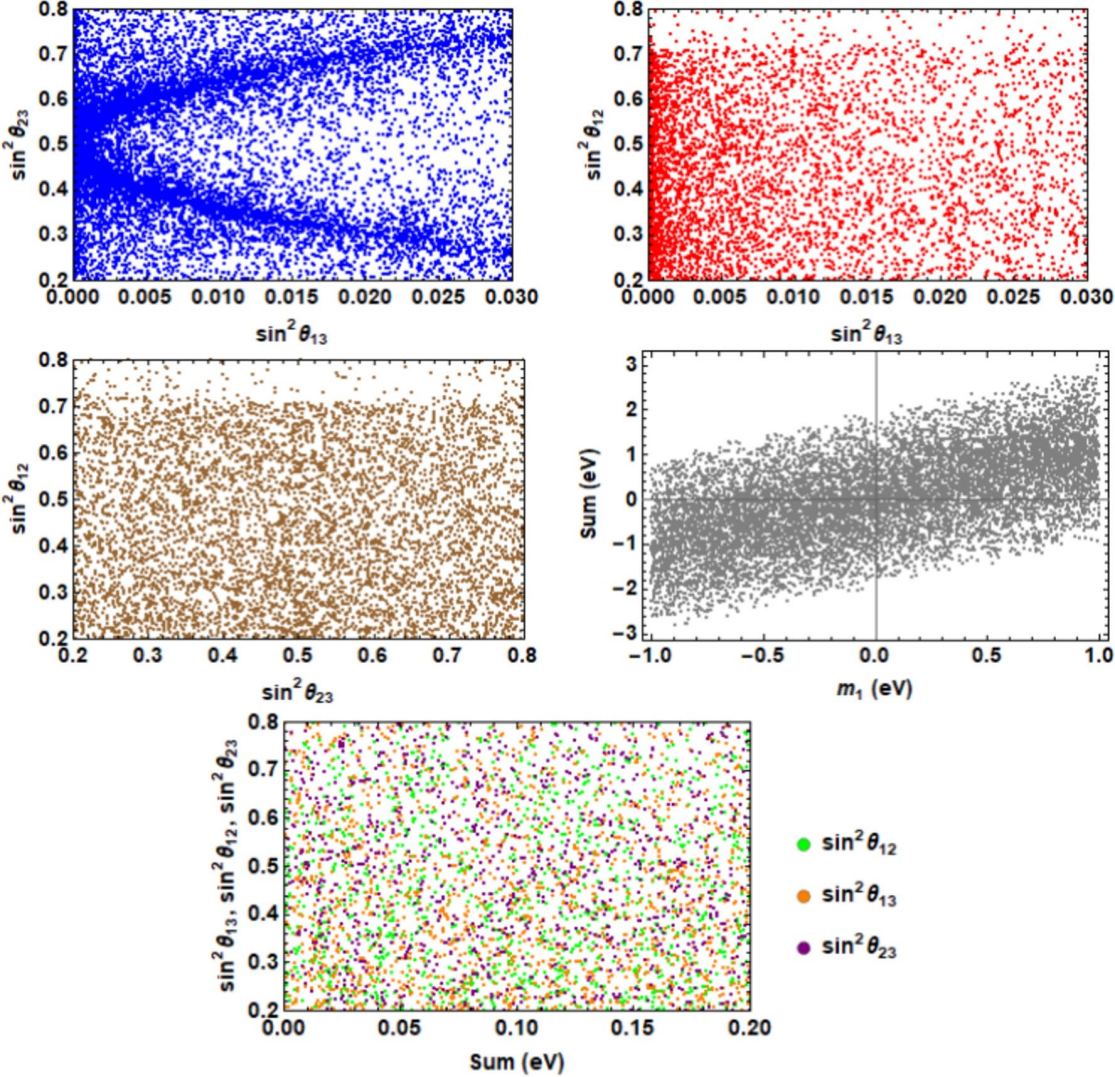
The correlation plots include $sin^2\theta _{13}$ versus $sin^2\theta _{23}$ and $sin^2\theta _{12}$, $sin^2\theta _{23}$ versus $sin^2\theta _{12}$, $m_1$ (neutrino mass in *eV* without perturbations) versus the sum of perturbed neutrino masses, and the sum of perturbed neutrino masses (in *eV*) versus $sin^2\theta _{13}$, $sin^2\theta _{12}$, and $sin^2\theta _{23}$ as shown in last plot.

## Numerical analysis

Taking into consideration the latest experimental data^26^, the mixing angles are measured as follows: The solar neutrino mixing angle, $\theta _{12}$, is determined to be $34^\circ \pm 1^\circ$, the atmospheric neutrino mixing angle, $\theta _{23}$, is found to be $42^\circ \pm 3^\circ$, and the reactor angle, $\theta _{13}$, is measured to be $8.5^\circ \pm 0.5^\circ$. Additionally, the squared mass differences are determined as $\Delta m_{\text {sol}}^2 = m_2^{{\prime }^2} - m_1^{{\prime }^2} \approx 7.53 \times 10^{-5}$ $\hbox {eV}^2$ and $\Delta m_{\text {atm}}^2 = m_3^{{\prime }^2} - m_2^{{\prime }^2} \approx 2.453 \times 10^{-3}$ $\hbox {eV}^2$ ($\Delta m_{\text {atm}}^2 = m_3^{{\prime }^2} - m_2^{{\prime }^2} \approx -2.536 \times 10^{-3}$ $\hbox {eV}^2$) for normal (inverted) neutrino mass ordering^97^. The lower and upper bounds of $\Sigma m^{\prime }$ are constrained to 0.06 eV and 0.12 eV, respectively^98^. Utilizing Eqs. (11) and (12), the objective or fitness function $(\epsilon )$ corresponding to these experimental constraints can be expressed as follows.

13 $$\begin{aligned} \begin{aligned} \epsilon =\epsilon _1+\epsilon _2+\epsilon _3+\epsilon _4+\epsilon _5+\epsilon _6, \end{aligned} \end{aligned}$$

with,

14 $$\begin{aligned} \begin{aligned} \epsilon _{1}&=\Biggl [{m^{\prime }_2}^2-{m^{\prime }}_1^2-\Delta \text {m}_{\text {sol}}^2\Biggl ]^2,\quad \epsilon _{2}=\Biggl [{m^{\prime }_3}^2-{m^{\prime }}_2^2-\Delta \text {m}_{\text {atm}}^2\Biggl ]^2,\quad \epsilon _{3}=\Biggl [\frac{|U_{12}|}{|U_{11}|}-t_{12}\Biggl ]^2,\\ \epsilon _{4}&=\Biggl [\frac{|U_{23}|}{|U_{33}|}-t_{23}\Biggl ]^2,\quad \epsilon _{5}=\Biggl [U_{13}-s_{13}\Biggl ]^2,\\ \epsilon _{6}&=\Biggl [m^{\prime }_1+m^{\prime }_2+m^{\prime }_3-\Biggl (\begin{array}{ll} 0.12\ eV,\quad \text {for upper bound limit} \\ 0.06\ eV,\quad \text {for lower bound limit} \\ \end{array}\Biggl )\Biggl ]^2, \end{aligned} \end{aligned}$$

where, $m^{\prime }_{1,2,3}$, $U_{11}$, $U_{12}$, $U_{23}$, $U_{33}$, $U_{13}$ are given in Eqs. (10) and (11).

In this article we use PSO instead of classical Chi-square fitting for optimization and parameter estimation tasks because of following reasons: (1) PSO is a population-based optimization technique that searches the entire solution space for the optimal solution. It can escape local optima more effectively compared to Chi-square fitting, which might get stuck in local minima. (2) PSO tends to converge faster than Chi-square fitting. (3) PSO does not require initial guesses for the parameters being optimized, unlike Chi-square fitting, where a good initial guess is required for convergence to the proper solution. (4) PSO can handle non-linear optimization problems efficiently, which may be challenging for Chi-square fitting. (5) PSO is highly adaptable and can be easily modified or extended to handle various types of optimization tasks, including parameter estimation, feature selection, and function optimization. (6) PSO can be parallelized effectively, allowing for faster computation times on parallel computing architectures compared to Chi-square fitting, which may not be as easily parallelized. (7) PSO is robust to noise and can handle noisy objective functions better than some Chi-square fitting methods, which may struggle with noisy data.

We use PSO algorithm to optimize the objective or fitness functions $\epsilon$ for optimal values of parameters. In the usual way of doing particle swarm optimization (PSO), we treat each possible solution to a problem is represented as a moving point within the search space. These points form a group called a ’swarm’, and they work together to check out the whole search area. Each point is assigned a unique score based on its efficacy in solving the problem. Initially, these points are randomly selected. Throughout each iteration, the positions and velocities of the points are updated based on their previous performance based on its local $P^{x-1}_{LB}$ and global $P^{x-1}_{GB}$ positions. The basic rules for updating position and velocity of a point are given as,

15 $$\begin{aligned} & \begin{aligned} v^{x}_i=wv^{t-1}_i+c_1 r_1(P^{x-1}_{LB}-X^{x-1}_{i})+c_2 r_2(P^{x-1}_{GB}-X^{x-1}_{i}), \end{aligned} \end{aligned}$$

16 $$\begin{aligned} & \begin{aligned} X^{x}_i=X^{x-1}_i+v^{x-1}_i. \end{aligned} \end{aligned}$$

In these rules, *i* goes from 1 to *p*, where *p* is just a integer telling us how many points there are. The weight ’*w*’ and $c_1$ and $c_2$ are also integers that help to control how the points move. Also, the velocity gradually gets smaller as we keep looking around (between 0 and 1). The random numbers $r_1$ and $r_2$ are just there to add a bit of randomness. Finally, the velocity of the points is kept within certain limits so they don’t go too fast or too slow.

The points traverse the search space by adapting their positions and velocities, drawing from their individual experiences and insights gained from neighboring points. The algorithm involves the following key steps:

1. Initialization: Commence by populating a set of points, assigning them random positions and velocities distributed throughout the exploration area.
2. Objective Assessment: Assess the fitness or objective function value for each point according to its present position.
3. Update Personal and Global Bests: Update the personal best position (Pbest) for each point based on its current fitness. Update the global best position (Gbest) considering the best position among all points.
4. Update Velocities and Positions: Adjust the velocity and position of each point using its current velocity, personal best, and global best positions.
5. Iteration: Continue steps 2 through 4 for a predetermined number of iterations or until reaching a convergence criterion.

**Fig. 2:**
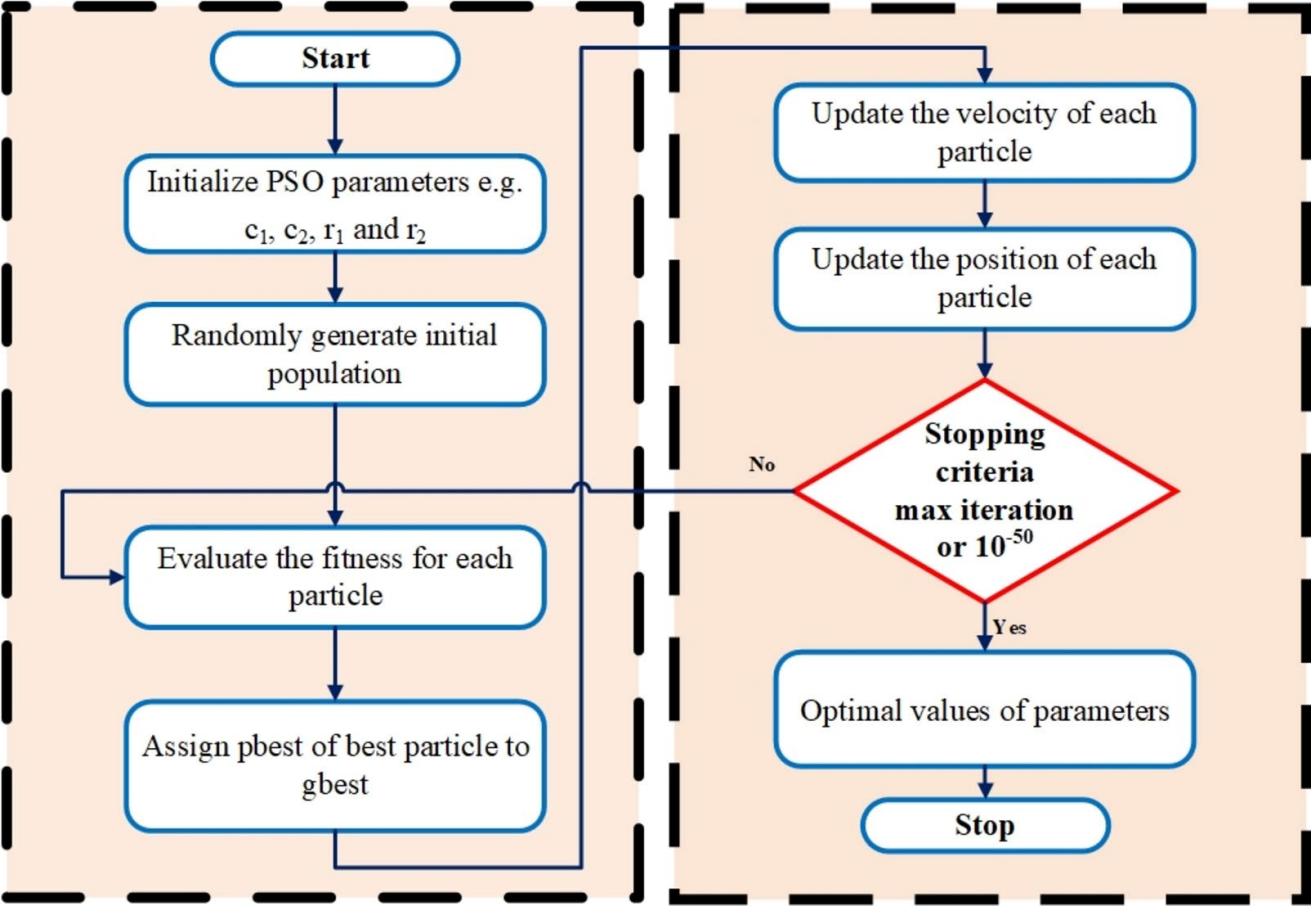
Generic flow chart of PSO.

The generic flow chart PSO is given in Fig. 2.

**Fig. 3:**
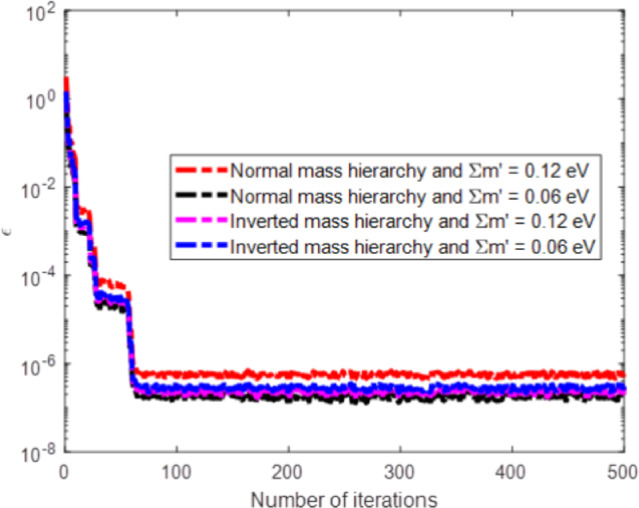
Fitness function versus number of iterations.

To inspire the development of meta-heuristic optimization algorithms, we employed PSO technique to minimize the objective function for both mass hierarchy and for upper and lower bound limits of $\Sigma m^{\prime }$. The objective function is minimized through PSO with 500 iteration are presented in Fig. 3 and corresponding values of *p*, $\theta$, $m_1$, $m_2$ and $m_3$ are given in Tables 2, 3, 4 and 5 in which $m_1$, $m_2$ and $m_3$ are masses of neutrinos in *eV* without perturbations correspond to zero reactor angle and *p* and $\theta$ are the free parameters of the model. The masses of neutrino without perturbations ($m_1$, $m_2$, $m_3$) and other model free parameters *p* and $\theta$ are predicted using PSO by using experimental squared mass differences of perturbed masses.

**Table 2:** The optimal values of parameters *p*, $\theta$, $m_1$, $m_2$, $m_3$, through PSO for upper bound limit of $\Sigma m^{\prime }=0.12\ eV$ and normal mass hierarchy.

Parameters	Optimal values	Parameters	Optimal values
*p*	$-4.5205\times 10^{-16}$	$\theta$	0.5894 *rad*
$m_1$	$4.0000\times 10^{-2}\ eV$	$m_2$	$4.0000\times 10^{-2}\ eV$
$m_3$	$4.0000\times 10^{-2}\ eV$

**Table 3:** The optimal values of parameters *p*, $\theta$, $m_1$, $m_2$, $m_3$, through PSO for lower bound limit of $\Sigma m^{\prime }=0.06\ eV$ and normal mass hierarchy.

Parameters	Optimal values	Parameters	Optimal values
*p*	$1.0964\times 10^{-14}$	$\theta$	0.5909 *rad*
$m_1$	$2.0000\times 10^{-2}\ eV$	$m_2$	$2.0000\times 10^{-2}\ eV$
$m_3$	$2.0000\times 10^{-2}\ eV$

**Table 4:** The optimal values of parameters *p*, $\theta$, $m_1$, $m_2$, $m_3$, through PSO for inverted mass hierarchy and for upper bound limit of $\Sigma m^{\prime }=0.12\ eV$.

Parameters	Optimal values	Parameters	Optimal values
*p*	$1.0000\times 10^{-4}$	$\theta$	0.5909 *rad*
$m_1$	$3.8627 \times 10^{-2}\ eV$	$m_2$	$4.0517\times 10^{-2}\ eV$
$m_3$	$3.8563\times 10^{-2}\ eV$

**Table 5:** The optimal values of parameters *p*, $\theta$, $m_1$, $m_2$, $m_3$, through PSO for lower bound limit of $\Sigma m^{\prime }=0.06\ eV$ and inverted mass hierarchy.

Parameters	Optimal values	Parameters	Optimal values
*p*	$1.4714\times 10^{-3}$	$\theta$	0.5909 *rad*
$m_1$	$1.1040\times 10^{-2}\ eV$	$m_2$	$3.8846\times 10^{-2}\ eV$
$m_3$	$1.0098\times 10^{-2}\ eV$		

The lepton mixing matrices upto second order perturbation theory for both mass hierarchy and for upper ($0.12\ eV$) and lower ($0.06\ eV$) bound limits of $\Sigma m^{\prime }$ are given as:

17 $$\begin{aligned}  &   \begin{aligned} |U^{(N)_{upper}}_{PMNS}|=\left( \begin{array}{ccc} 0.8208 &  0.5519 &  0.1476 \\ 0.3317 &  0.6710 &  0.6617 \\ 0.4653 &  0.4952 &  0.7353 \\ \end{array} \right) \end{aligned} \end{aligned}$$

18 $$\begin{aligned}  &   \begin{aligned} |U^{(N)_{lower}}_{PMNS}|=\left( \begin{array}{ccc} 0.8201 &  0.5539 &  0.1470 \\ 0.3336 &  0.6710 &  0.6615 \\ 0.4650 &  0.4938 &  0.7355 \\ \end{array} \right) \end{aligned} \end{aligned}$$

19 $$\begin{aligned}  &   \begin{aligned} |U^{(I)_{upper}}_{PMNS}|=\left( \begin{array}{ccc} 0.8200 &  0.5531 &  0.1477 \\ 0.3330 &  0.6710 &  0.6618 \\ 0.4657 &  0.4938 &  0.7350 \\ \end{array} \right) \end{aligned} \end{aligned}$$

20 $$\begin{aligned}  &   \begin{aligned} |U^{(I)_{lower}}_{PMNS}|=\left( \begin{array}{ccc} 0.8200 &  0.5531 &  0.1478 \\ 0.3330 &  0.6710 &  0.6618 \\ 0.4658 &  0.4938 &  0.7350 \\ \end{array} \right) \end{aligned} \end{aligned}$$

On the behalf of the values of *p*, $$\theta, $$ $$m_1,$$ $$m_2$$ and $$m_3,$$ the mass corrections upto second order perturbation theory for both mass hierarchy and for upper ($$0.12\ eV$$) and lower ($$0.06\ eV$$) bound limits of $$\Sigma m^{\prime }$$ are given as: $${m_{1}^{\prime }}^{(N)}(upper)=4.0000 \times 10^{-2}\ eV,$$ $${m_{2}^{\prime }}^{(N)}(upper)=4.0000 \times 10^{-2}\ eV,$$ $${m_{3}^{\prime }}^{(N)}(upper)=4.0000 \times 10^{-2}\ eV, {m_{1}^{\prime }}^{(I)}(upper)$$ $$=3.8628\times 10^{-2}\ eV, {m_{2}^{\prime }}^{(I)}(upper)=4.0548\times 10^{-2}\ eV, {m_{3}^{\prime }}^{(I)}(upper)=3.8532\times 10^{-2}\ eV,$$$${m_{1}^{\prime }}^{(N)}(lower)=2.0000 \times 10^{-2}\ eV, {m_{2}^{\prime }}^{(N)}(lower)=2.0000 \times 10^{-2}\ eV,$$ $${m_{3}^{\prime }}^{(N)}(lower)=2.0000 \times 10^{-2}\ eV, {m_{1}^{\prime }}^{(I)}(lower)$$ $$=1.1049\times 10^{-2}\ eV, {m_{2}^{\prime }}^{(I)}(lower)=3.9298\times 10^{-2}\ eV$$ and $${m_{3}^{\prime }}^{(I)}(lower)=9.6381\times 10^{-3}\ eV,$$ which are correspond to non zero reactor angle. The Dirac CP-violating phase $$\delta $$ is measured to be $$-\pi /2$$. In our numerical analysis, we concentrated on only two specific values for the sum of neutrino masses, instead of a continuous spectrum. We utilized 0.06 eV, representing the lower limit of the sum of masses, and 0.12 eV as the upper limit. In the NO case and the neutrino masses appear nearly degenerate and normal hierarchy could not be adequately reproduced here. In contrast, for the inverted ordering, a more defined hierarchy was observable, making the model consistent with an inverted hierarchy (IO). These findings are consistent with a quasi-degenerate normal hierarchy (QDNH) mass spectrum, characterized by $${m_{1}^{\prime }}^N\lesssim {m_{2}^{\prime }}^N\lesssim {m_{3}^{\prime }}^N,$$ and an inverted mass hierarchy (IH), where $${m_{3}^{\prime }}^I<{m_{1}^{\prime }}^I<{m_{2}^{\prime }}^I$$ for both $$\Sigma m^{\prime }=0.06$$ and 0.12.

### Effective neutrino mass parameters

The expressions for the effective neutrino masses^99–103^ associated with neutrinoless double beta decay ($\langle m_{ee} \rangle$) and beta decay ($m_\beta$) are structured as follows:

21 $$\begin{aligned} \begin{aligned} m_{\beta } =\sqrt{\sum _{i = 1}^{3}{|U_{\text {ei}}|^{2}m_{i}^{{\prime }^2}}}, \quad \left\langle m_{\text {ee}} \right\rangle = \left| \sum _{i = 1}^{3}{U_{\text {ei}}^{2}m_{i}^{\prime }} \right| \end{aligned}, \end{aligned}$$

Considering the leptonic mixing matrix elements $U_{ei}$ with *i* ranging from 1 to 3, representing the masses $m^{\prime }_i$ of three neutrinos, the effective neutrino masses associated with neutrinoless double beta decay ($\langle m_{ee} \rangle$) and beta decay ($m_\beta$) are computed using the parameters obtained in section 3. This calculation is performed for both mass hierarchy of neutrino, yielding the following results:

22 $$\begin{aligned} \begin{aligned} \langle {m_{ee}} \rangle ^{N}(upper)&=40.0050 \ meV, \qquad \langle {m_{\beta }} \rangle ^{N}(upper)&=40.0025\ meV,\\ \langle {m_{ee}} \rangle ^{I}(upper)&=39.2181\ meV, \qquad \langle {m_{\beta }} \rangle ^{I}(upper)&=39.2257\ meV,\\ \langle {m_{ee}} \rangle ^{N}(lower)&=20.0024\ meV, \qquad \langle {m_{\beta }} \rangle ^{N}(lower)&=20.0012\ meV,\\ \langle {m_{ee}} \rangle ^{I}(lower)&=19.6608\ meV, \qquad \langle {m_{\beta }} \rangle ^{I}(lower)&=23.5908\ meV. \end{aligned} \end{aligned}$$

## Vacuum alignment studies

In particle physics, the dynamics of scalar fields are encapsulated by an invariant scalar potential within the symmetry group. The following Eq. (23) describes the invariant scalar potential within the symmetry group $SU(2)_L \times U(1)_Y \times A_{4}\times Z_{10} \times Z_{3}$. It plays a crucial role in understanding spontaneous symmetry breaking and the generation of particle masses. While $A_4$, $Z_{10}$ and $Z_{3}$ are discrete symmetries that add to the rich structure of the potential, the $SU(2)_L$ symmetry describes weak isospin.

23 $$\begin{aligned} \begin{aligned} V=&V(\phi )+ V(\Phi )+V(\Delta )+V(\eta )+ V(\kappa )+V(\xi )+V({\xi ^{\prime }})+V(\phi ,\Phi )+V(\phi , \Delta )+V(\phi ,\eta )\\ &+V(\phi ,\kappa )+V(\phi ,\xi )+V(\phi ,{\xi ^{\prime }})+V(\Phi , \Delta )+V(\Phi ,\eta )+V(\Phi ,\kappa )+V(\Phi ,\xi )+V(\Phi ,{\xi ^{\prime }})\\ &+V(\Delta ,\eta )+V(\Delta ,\kappa )+V(\Delta ,\xi )+V(\Delta ,{\xi ^{\prime }})+V(\eta ,\kappa )+V(\eta ,\xi )+V(\eta ,{\xi ^{\prime }})+V(\kappa ,\xi )\\ &+V(\kappa ,{\xi ^{\prime }})+V(\xi ,{\xi ^{\prime }})+V(\phi ,\eta ,\kappa )+V(\phi ,\xi ,{\xi ^{\prime }})+ V(\Phi ,\eta ,\kappa )+V(\Phi ,\xi ,{\xi ^{\prime }})+V(\Delta ,\eta ,\kappa ) \\ &+V(\Delta ,\xi ,{\xi ^{\prime }})+V(\eta ,\kappa ,\xi ,{\xi ^{\prime }}). \end{aligned} \end{aligned}$$

Writing the terms explicitly, we have,

24 $$\begin{aligned} V(\phi )&=-\mu ^2_{\phi }(\phi ^{\dagger }\phi )+ \lambda ^{\phi }_1(\phi ^{\dagger }\phi )(\phi ^{\dagger }\phi )+\lambda ^{\phi }_2(\phi ^{\dagger }\phi )_{1'}(\phi ^{\dagger }\phi )_{1''}+\lambda ^{\phi }_3(\phi ^{\dagger }\phi )_{3_s}(\phi ^{\dagger }\phi )_{3_s},\nonumber \\ V(\Phi )&= V(\phi \rightarrow \Phi ),\nonumber \\ V(\Delta )&=-\mu ^2_{\Delta }Tr(\Delta ^{\dagger }\Delta )+ \lambda ^{\Delta }_1 Tr(\Delta ^{\dagger }\Delta )Tr(\Delta ^{\dagger }\Delta )+\lambda ^{\Delta }_2 Tr(\Delta ^{\dagger }\Delta )_{1'} Tr(\Delta ^{\dagger }\Delta )_{1''}\nonumber \\&\quad \,+\lambda ^{\Delta }_3 Tr(\Delta ^{\dagger }\Delta )_{3_s} Tr(\Delta ^{\dagger }\Delta )_{3_s}\nonumber \\ V(\eta )&= -\mu ^2_{\eta }(\eta ^{*}\eta )+ \lambda ^{\eta }(\eta ^{*}\eta )^2,\quad V(\kappa )= -\mu ^2_{\kappa }(\kappa ^{*}\kappa )+ \lambda ^{\kappa }(\kappa ^{*}\kappa )^2,\nonumber \\ V(\xi )&= -\mu ^2_{\xi }(\xi ^{*}\xi )+ \lambda ^{\xi }(\xi ^{*}\xi )^2,\quad V({\xi ^{\prime }})= -\mu ^2_{{\xi ^{\prime }}}({\xi ^{\prime }}^{*}{\xi ^{\prime }})+ \lambda ^{{\xi ^{\prime }}}({\xi ^{\prime }}^{*}{\xi ^{\prime }})^2,\nonumber \\ V(\phi ,\Phi )&=\lambda ^{\phi \Phi }_1(\phi ^{\dagger }\phi )(\Phi ^{\dagger }\Phi )+\lambda ^{\phi \Phi }_2((\phi ^{\dagger }\phi )_{1'}(\Phi ^{\dagger }\Phi )_{1''}+(\phi ^{\dagger }\phi )_{1''}(\Phi ^{\dagger }\Phi )_{1'})\nonumber \\ &+\lambda ^{\phi \Phi }_3(\phi ^{\dagger }\phi )_{3_s}(\Phi ^{\dagger }\Phi )_{3_s},\nonumber \\ V(\phi , \Delta )&= V(\phi , \Phi \rightarrow \phi ,\Delta ),\quad V(\phi ,\eta )=\lambda ^{\phi \eta }(\phi ^{\dagger }\phi )\eta ^{*}\eta , \quad V(\phi ,\kappa )=\lambda ^{\phi \kappa }(\phi ^{\dagger }\phi )\kappa ^{*}\kappa ,\nonumber \\ V(\phi ,\xi )&=\lambda ^{\phi \xi }(\phi ^{\dagger }\phi )\xi ^{*}\xi ,\quad V(\phi ,{\xi ^{\prime }})=\lambda ^{\phi {\xi ^{\prime }}}(\phi ^{\dagger }\phi ){\xi ^{\prime }}^{*}{\xi ^{\prime }},\quad V(\Phi ,\Delta )=V(\phi ,\Phi \rightarrow \Phi ,\Delta ),\nonumber \\ V(\Phi ,\eta )&=\lambda ^{\Phi \eta }(\Phi ^{\dagger }\Phi )\eta ^{*}\eta ,\quad V(\Phi ,\kappa )=\lambda ^{\Phi \kappa }(\Phi ^{\dagger }\Phi )\kappa ^{*}\kappa ,\quad V(\Phi ,\xi )=\lambda ^{\Phi \xi }(\Phi ^{\dagger }\Phi )\xi ^{*}\xi ,\nonumber \\ V(\Phi ,{\xi ^{\prime }})&=\lambda ^{\Phi {\xi ^{\prime }}}(\Phi ^{\dagger }\Phi ){\xi ^{\prime }}^{*}{\xi ^{\prime }},\quad V(\Delta ,\eta )=\lambda ^{\eta \Delta }Tr(\Delta ^{\dagger }\Delta )\eta ^{*}\eta ,\quad V(\Delta ,\kappa )=\lambda ^{\kappa \Delta }Tr(\Delta ^{\dagger }\Delta )\kappa ^{*}\kappa ,\nonumber \\ V(\Delta ,\xi )&=\lambda ^{\Delta \xi }Tr(\Delta ^{\dagger }\Delta )\xi ^{*}\xi ,\quad V(\Delta ,{\xi ^{\prime }})=\lambda ^{\Delta {\xi ^{\prime }}}Tr(\Delta ^{\dagger }\Delta ){\xi ^{\prime }}^{*}{\xi ^{\prime }},\nonumber \\ V(\eta ,\kappa )&=\lambda ^{\eta \kappa }(\eta ^{*}\eta \kappa ^{*}\kappa +\mu ^2_{\eta \kappa }(\eta \kappa +h.c)+(\eta ^2 \kappa ^2+h.c)),\nonumber \\ V(\eta ,\xi )&=\lambda ^{\eta \xi }\eta ^{*}\eta \xi ^{*}\xi ,\quad V(\eta ,{\xi ^{\prime }})=\lambda ^{\eta {\xi ^{\prime }}}\eta ^{*}\eta {\xi ^{\prime }}^{*}{\xi ^{\prime }},\quad V(\xi ,{\xi ^{\prime }})=V(\eta , \kappa \rightarrow \xi , {\xi ^{\prime }}),\nonumber \\V(\kappa ,\xi )&=\lambda ^{\kappa \xi }\kappa ^{*}\kappa \xi ^{*}\xi ,\quad V(\kappa ,{\xi ^{\prime }})=\lambda ^{\kappa {\xi ^{\prime }}}\kappa ^{*}\kappa {\xi ^{\prime }}^{*}{\xi ^{\prime }},\quad V(\phi ,\eta ,\kappa )=\lambda ^{\phi \eta \kappa }((\phi ^{\dagger }\phi )\eta \kappa +h.c),\nonumber \\ V(\phi ,\xi ,{\xi ^{\prime }})&=\lambda ^{\phi \xi {\xi ^{\prime }}}((\phi ^{\dagger }\phi )\xi {\xi ^{\prime }}+h.c),\quad V(\Phi ,\eta ,\kappa )=\lambda ^{\Phi \eta \kappa }((\Phi ^{\dagger }\Phi )\eta \kappa +h.c),\nonumber \\ V(\Phi ,\xi ,{\xi ^{\prime }})&=\lambda ^{\Phi \xi {\xi ^{\prime }}}((\Phi ^{\dagger }\Phi )\xi {\xi ^{\prime }}+h.c),\quad V(\Delta ,\eta ,\kappa )=\lambda ^{\Delta \eta \kappa }(Tr(\Delta ^{\dagger }\Delta )\eta \kappa +h.c),\nonumber \\ V(\Delta ,\xi ,{\xi ^{\prime }})&=\lambda ^{\Delta \xi {\xi ^{\prime }}}(Tr(\Delta ^{\dagger }\Delta )\xi {\xi ^{\prime }}+h.c),\quad V(\eta ,\kappa ,\xi ,{\xi ^{\prime }})=\lambda ^{\eta \kappa \xi {\xi ^{\prime }}}(\eta \kappa \xi {\xi ^{\prime }}+h.c). \end{aligned}$$

Many terms containing $(\phi ^{\dagger }\phi )_{3_a},\ (\Phi ^{\dagger }\Phi )_{3_a}$ and $Tr(\Delta ^{\dagger }\Delta )_{3_a}$ are also invariant but are not appeared in (23) because they are vanished due to the antisymmetric of $3_a$ as a consequence of the tensor product of $3\times 3$ of $A_4$ and the VEV alignment of $\phi$, $\Phi$ and $\Delta$. The minimization conditions (VEVs) of this potential can result in the extreme solutions detailed in (25). These VEVs provide information about the stable configurations of the system since they represent crucial places where potential energy is minimized.

25 $$\begin{aligned} \begin{aligned} \left\langle \phi \right\rangle&= (\langle \phi _1\rangle ,\quad 0,\quad 0), \quad \langle \phi _1\rangle =(0 \quad v)^{T},\quad \langle \Phi \rangle = (0,\quad \langle \Phi _2\rangle ,\quad \langle \Phi _3 \rangle ),\\ \quad \langle \Phi _2\rangle&= \langle \Phi _3\rangle =(u \quad 0)^{T},\quad \langle \Delta \rangle = (0,\quad \langle \Delta _2\rangle ,\quad \langle \Delta _3 \rangle ),\\ \quad \langle \Delta _2\rangle&= \begin{pmatrix} 0 & 0 \\ w & 0\\ \end{pmatrix}, \quad \langle \Delta _3\rangle = -\begin{pmatrix} 0 & 0 \\ w & 0\\ \end{pmatrix}, \quad \left\langle \eta \right\rangle =\left\langle \kappa \right\rangle =v_m, \quad \left\langle \xi \right\rangle =\left\langle \xi ^{\prime } \right\rangle =v_\epsilon , \end{aligned} \end{aligned}$$

**PSO Treatment for Scalar Potential:**

Due to VEVs (24), multiplication rules for $A_4$ symmetry in the T-diagonal basis^104^ and $S_2$ symmetry, Eq. (23) become as

26 $$\begin{aligned} \begin{aligned} V=&-\mu ^2_{\phi }v^2+\lambda ^{\phi }_{1}v^4+\frac{4}{9}\lambda ^{\phi }_{3}v^4-2\mu ^2_{\Phi }u^2+4\lambda ^{\Phi }_{1}u^4+\lambda ^{\Phi }_{2}u^4+\frac{12}{9}\lambda ^{\Phi }_{3}u^4+2\mu ^2_{\Delta }w^2+4\lambda ^{\Delta }_1w^4\\ &+\lambda ^{\Delta }_2w^4+\frac{12}{9}\lambda ^{\Delta }_3w^4+2\lambda ^{\phi \Phi }_{1}v^2u^2-\frac{4}{9}\lambda ^{\phi \Phi }_{3}v^2u^2-2\lambda ^{\phi \Delta }_1v^2w^2+\frac{4}{9}\lambda ^{\phi \Delta }_3v^2w^2-4\lambda ^{\Phi \Delta }_1u^2w^2\\ &+2\lambda ^{\Phi \Delta }_2u^2w^2+\frac{4}{9}\lambda ^{\Phi \Delta }_3u^2w^2-\mu ^2_\eta v^2_m+\lambda ^{\eta }v^4_m-\mu ^2_\kappa v^2_m+\lambda ^{\kappa }v^4_m-\mu ^2_\xi v^2_\epsilon +\lambda ^{\xi }v^4_\epsilon -\mu ^2_{\xi ^{\prime }} v^2_\epsilon \\ &+\lambda ^{{\xi ^{\prime }}}v^4_\epsilon +\lambda ^{\phi \eta }v^2v^2_m+\lambda ^{\phi \kappa }v^2v^2_m+\lambda ^{\phi \xi }v^2v^2_\epsilon +\lambda ^{\phi \xi ^{\prime }}v^2v^2_\epsilon +2\lambda ^{\Phi \eta }u^2v^2_m+2\lambda ^{\Phi \kappa }u^2v^2_m\\ &+2\lambda ^{\Phi \xi }u^2v^2_\epsilon +2\lambda ^{\Phi \xi ^{\prime }}u^2v^2_\epsilon -2\lambda ^{\eta \Delta }w^2v^2_m-2\lambda ^{\kappa \Delta }w^2v^2_m-2\lambda ^{\Delta \xi }w^2v^2_\epsilon -2\lambda ^{\Delta \xi ^{\prime }}w^2v^2_\epsilon \\ &+\lambda ^{\eta \kappa }(v^4_m+2\mu ^2_{\eta \kappa }v^2_m+2v^4_m)+\lambda ^{\eta \xi }v^2_m v^2_\epsilon +\lambda ^{\eta \xi ^{\prime }}v^2_m v^2_\epsilon +\lambda ^{\xi \xi ^{\prime }}(v^4_\epsilon +2\mu ^2_{\xi \xi ^{\prime }}v^2_\epsilon +2v^4_\epsilon )\\ &+\lambda ^{\kappa \xi }v^2_mv^2_\epsilon +\lambda ^{\kappa \xi ^{\prime }}v^2_m v^2_\epsilon +2\lambda ^{\phi \eta \kappa }v^2 v^2_m+2\lambda ^{\phi \xi \xi ^{\prime }}v^2 v^2_\epsilon +4\lambda ^{\Phi \eta \kappa }u^2v^2_m+4\lambda ^{\Phi \xi \xi ^{\prime }}u^2v^2_\epsilon \\ &-4\lambda ^{\Delta \eta \kappa }w^2v^2_m-4\lambda ^{\Delta \xi \xi ^{\prime }}w^2v^2_\epsilon +2\lambda ^{\eta \kappa \xi \xi ^{\prime }}v^2_mv^2_\epsilon , \end{aligned} \end{aligned}$$

and the fitness function $(\epsilon ^{\prime })$ for (26) is expressed as follows.

27 $$\begin{aligned} \begin{aligned} \epsilon ^{\prime }=\biggl [&-\mu ^2_{\phi }v^2+\lambda ^{\phi }_{1}v^4+\frac{4}{9}\lambda ^{\phi }_{3}v^4-2\mu ^2_{\Phi }u^2+4\lambda ^{\Phi }_{1}u^4+\lambda ^{\Phi }_{2}u^4+\frac{12}{9}\lambda ^{\Phi }_{3}u^4+2\mu ^2_{\Delta }w^2+4\lambda ^{\Delta }_1w^4\\ &+\lambda ^{\Delta }_2w^4+\frac{12}{9}\lambda ^{\Delta }_3w^4+2\lambda ^{\phi \Phi }_{1}v^2u^2-\frac{4}{9}\lambda ^{\phi \Phi }_{3}v^2u^2-2\lambda ^{\phi \Delta }_1v^2w^2+\frac{4}{9}\lambda ^{\phi \Delta }_3v^2w^2-4\lambda ^{\Phi \Delta }_1u^2w^2\\ &+2\lambda ^{\Phi \Delta }_2u^2w^2+\frac{4}{9}\lambda ^{\Phi \Delta }_3u^2w^2-\mu ^2_\eta v^2_m+\lambda ^{\eta }v^4_m-\mu ^2_\kappa v^2_m+\lambda ^{\kappa }v^4_m-\mu ^2_\xi v^2_\epsilon +\lambda ^{\xi }v^4_\epsilon -\mu ^2_{\xi ^{\prime }} v^2_\epsilon \\ &+\lambda ^{{\xi ^{\prime }}}v^4_\epsilon +\lambda ^{\phi \eta }v^2v^2_m+\lambda ^{\phi \kappa }v^2v^2_m+\lambda ^{\phi \xi }v^2v^2_\epsilon +\lambda ^{\phi \xi ^{\prime }}v^2v^2_\epsilon +2\lambda ^{\Phi \eta }u^2v^2_m+2\lambda ^{\Phi \kappa }u^2v^2_m\\ &+2\lambda ^{\Phi \xi }u^2v^2_\epsilon +2\lambda ^{\Phi \xi ^{\prime }}u^2v^2_\epsilon -2\lambda ^{\eta \Delta }w^2v^2_m-2\lambda ^{\kappa \Delta }w^2v^2_m-2\lambda ^{\Delta \xi }w^2v^2_\epsilon -2\lambda ^{\Delta \xi ^{\prime }}w^2v^2_\epsilon \\ &+\lambda ^{\eta \kappa }(v^4_m+2\mu ^2_{\eta \kappa }v^2_m+2v^4_m)+\lambda ^{\eta \xi }v^2_m v^2_\epsilon +\lambda ^{\eta \xi ^{\prime }}v^2_m v^2_\epsilon +\lambda ^{\xi \xi ^{\prime }}(v^4_\epsilon +2\mu ^2_{\xi \xi ^{\prime }}v^2_\epsilon +2v^4_\epsilon )\\ &+\lambda ^{\kappa \xi }v^2_mv^2_\epsilon +\lambda ^{\kappa \xi ^{\prime }}v^2_m v^2_\epsilon +2\lambda ^{\phi \eta \kappa }v^2 v^2_m+2\lambda ^{\phi \xi \xi ^{\prime }}v^2 v^2_\epsilon +4\lambda ^{\Phi \eta \kappa }u^2v^2_m+4\lambda ^{\Phi \xi \xi ^{\prime }}u^2v^2_\epsilon \\ &-4\lambda ^{\Delta \eta \kappa }w^2v^2_m-4\lambda ^{\Delta \xi \xi ^{\prime }}w^2v^2_\epsilon +2\lambda ^{\eta \kappa \xi \xi ^{\prime }}v^2_mv^2_\epsilon \biggl ]^{2}, \end{aligned} \end{aligned}$$

**Fig. 4:**
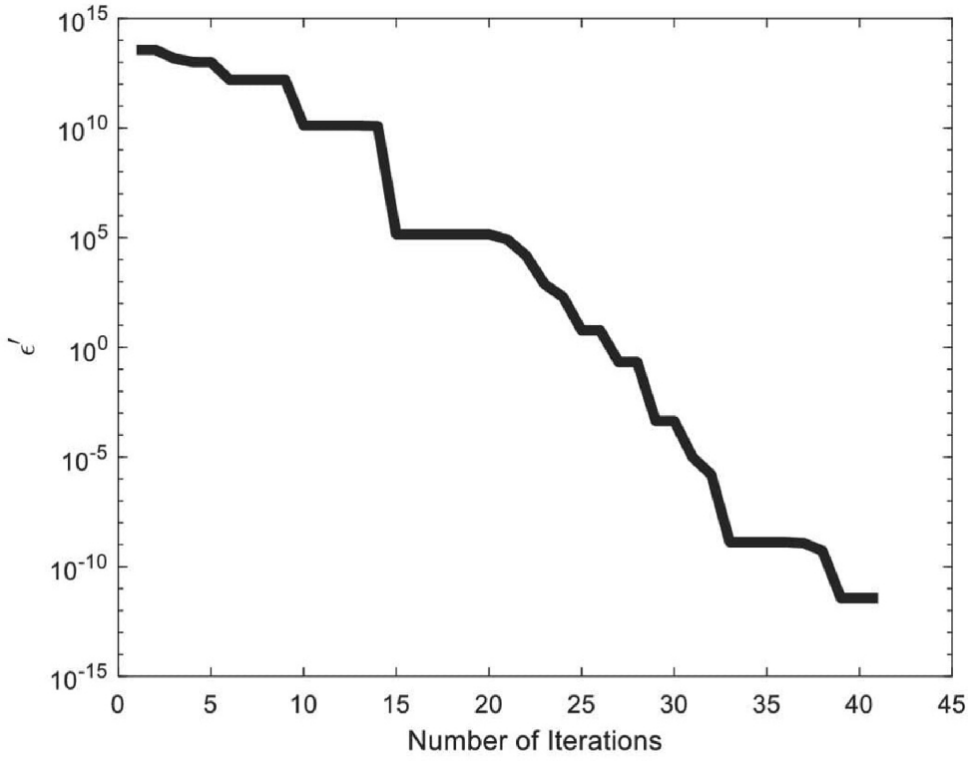
Fitness function of scalar potential versus number of iterations.

To stimulate the advancement of meta-heuristic optimization algorithms, we again utilized PSO technique to minimize the scalar potential. The objective function is minimized through PSO for scalar potential with 500 iteration is presented in Fig. 4.

This figure demonstrates that the objective function converges to zero with each iteration when employing the VEVs provided in (25). The optimal parameter values, as measured within the objective function of the scalar potential using PSO technique, are listed in Table 6.

**Table 6:** The optimal values of parameters given in (23) through PSO.

Parameters	Optimal values	Parameters	Optimal values
$\mu _\phi$	$179.175 \ GeV$	$\mu _\Phi$	$139.331 \ GeV$
$\mu _\Delta$	$145.538 \ GeV$	$\mu _\eta$	$178.33 \ GeV$
$\mu _\kappa$	$115.583 \ GeV$	$\mu _\xi$	$187.052 \ GeV$
$\mu _{\xi ^{\prime }}$	$153.417 \ GeV$	$\mu _{\eta \kappa }$	$104.519 \ GeV$
$\mu _{\xi \xi ^{\prime }}$	$158.023 \ GeV$	*v*	$134.316 \ GeV$
*u*	$113.798 \ GeV$	*w*	$134.684 \ GeV$
$v_m$	$158.347 \ GeV$	$v_\epsilon$	$159.224 \ GeV$
$\lambda ^{\phi }_1$	10.00000	$\lambda ^{\phi }_3$	6.08711
$\lambda ^{\Phi }_1$	$-8.95786$	$\lambda ^{\Phi }_2$	3.55938
$\lambda ^{\Phi }_3$	$-2.54363$	$\lambda ^{\Delta }_1$	$-2.6438$
$\lambda ^{\Delta }_2$	3.96077	$\lambda ^{\Delta }_3$	2.64742
$\lambda ^{\phi \Phi }_1$	9.7041	$\lambda ^{\phi \Phi }_3$	0.225504
$\lambda ^{\phi \Delta }_1$	7.95572	$\lambda ^{\phi \Delta }_3$	$-6.47177$
$\lambda ^{\Phi \Delta }_1$	$-0.953689$	$\lambda ^{\Phi \Delta }_2$	3.49581
$\lambda ^{\Phi \Delta }_3$	$-5.0444$	$\lambda ^\eta$	$-10.00000$
$\lambda ^\kappa$	9.14604	$\lambda ^\xi$	$-2.93147$
$\lambda ^{\xi ^{\prime }}$	$-2.35666$	$\lambda ^{\phi \eta }$	10.00000
$\lambda ^{\phi \kappa }$	8.98456	$\lambda ^{\phi \xi }$	$-10.00000$
$\lambda ^{\phi \xi ^{\prime }}$	1.73548	$\lambda ^{\Phi \eta }$	$-5.4436$
$\lambda ^{\Phi \kappa }$	$-4.29061$	$\lambda ^{\Phi \xi }$	$-3.53999$
$\lambda ^{\Phi \xi ^{\prime }}$	1.25844	$\lambda ^{\eta \Delta }$	$-4.9442$
$\lambda ^{\kappa \Delta }$	$-9.23625$	$\lambda ^{\Delta \xi }$	$-2.61907$
$\lambda ^{\Delta \xi ^{\prime }}$	$-5.5742$	$\lambda ^{\eta \kappa }$	10.00000
$\lambda ^{\eta \xi }$	$-1.84958$	$\lambda ^{\eta \xi ^{\prime }}$	2.21404
$\lambda ^{\xi \xi ^{\prime }}$	2.37551	$\lambda ^{\kappa \xi }$	10.00000
$\lambda ^{\kappa \xi ^{\prime }}$	$-7.90574$	$\lambda ^{\phi \eta \kappa }$	$-10.00000$
$\lambda ^{\phi \xi \xi ^{\prime }}$	$-0.973407$	$\lambda ^{\Phi \eta \kappa }$	$-2.65544$
$\lambda ^{\Phi \xi \xi ^{\prime }}$	$-10.00000$	$\lambda ^{\Delta \eta \kappa }$	$-1.32635$
$\lambda ^{\Delta \xi \xi ^{\prime }}$	7.21468	$\lambda ^{\eta \kappa \xi \xi ^{\prime }}$	1.23132

The scalar potential is minimized from these optimal values. In contrast to^95^, which analyzed the scalar potential without determining the masses of extra flavon fields, the PSO approach not only analyzes the scalar potential but also determines the numerical values of the flavon field masses. Table 6, revealing that the flavon masses are on the order of $O(100\ GeV)$, which is relevant to current experimental searches.

## Conclusion

In this study, we have examined a model within $SU(2)_L \times U(1)_Y \times A_{4}\times S_2\times Z_{10} \times Z_{3}$ to estimate the neutrino masses using particle swarm optimization technique for both neutrino hierarchy. In this model, a hybrid seesaw mechanism proposed for improved mass suppression and new mixing patterns by combining type-I and type-II and generate effective Majorana neutrino mass matrices. After calculating the mass eigenvalues and lepton mixing matrix upto second order perturbation theory in the framework based on $A_{4}$ symmetry, we investigated the minimization of the scalar potential for VEVs through PSO. The utilization of PSO in determining optimal parameters for computing $U_{PMNS}$ matrices, neutrino masses as: ${m_{1}^{\prime }}^{(N)}(upper)=4.0000 \times 10^{-2}\ eV,$ ${m_{2}^{\prime }}^{(N)}(upper)=4.0000 \times 10^{-2}\ eV, {m_{3}^{\prime }}^{(N)}(upper)$ $=4.0000 \times 10^{-2}\ eV, {m_{1}^{\prime }}^{(I)}(upper)=3.8628\times 10^{-2}\ eV,$ ${m_{2}^{\prime }}^{(I)}(upper)=4.0548\times 10^{-2}\ eV, {m_{3}^{\prime }}^{(I)}(upper)=3.8532\times 10^{-2}\ eV, {m_{1}^{\prime }}^{(N)}(lower)$ $=2.0000 \times 10^{-2}\ eV, {m_{2}^{\prime }}^{(N)}(lower)=2.0000 \times 10^{-2}\ eV, {m_{3}^{\prime }}^{(N)}(lower)$ $=2.0000 \times 10^{-2}\ eV, {m_{1}^{\prime }}^{(I)}(lower)=1.1049\times 10^{-2}\ eV, {m_{2}^{\prime }}^{(I)}(lower)=3.9298\times 10^{-2}\ eV$ and ${m_{3}^{\prime }}^{(I)}(lower)=9.6381\times 10^{-3}\ eV,$ effective neutrino mass parameters as: $\langle {m_{ee}} \rangle ^{N}(upper)=40.0050 \ meV,$ $\langle {m_{\beta }} \rangle ^{N}(upper)=40.0025\ meV,$ $\langle {m_{ee}} \rangle ^{I}(upper)=39.2181\ meV,$ $\langle {m_{\beta }} \rangle ^{I}(upper)=39.2257\ meV,$ $\langle {m_{ee}} \rangle ^{N}(lower)=20.0024\ meV,$ $\langle {m_{\beta }} \rangle ^{N}(lower)=20.0012\ meV,$ $\langle {m_{ee}} \rangle ^{I}(lower)=19.6608\ meV,$ $\langle {m_{\beta }} \rangle ^{I}(lower)=23.5908\ meV$ for both mass hierarchy are illustrated as well. The Dirac CP-violating phase $\delta$ is measured to be $-\pi /2$.

## Data Availability

The datasets used and/or analysed during the current study available from the corresponding author on reasonable request.
